# The effect of physiotherapy in rotator cuff injury patients with platelet-rich plasma: study protocol of a non-randomized controlled trial

**DOI:** 10.1186/s12891-021-04171-2

**Published:** 2021-03-20

**Authors:** Chi Zhang, Jianxiong Wang, Li Wang, Yujie Xie, Fuhua Sun, Wei Jiang, Akira Miyamoto, Lei Lei

**Affiliations:** 1 Department of Rehabilitation, The Affiliated Hospital of Southwest Medical University, 25 Tai Ping Road, Luzhou, Sichuan 646000 People’s Republic of China; 2 Rehabilitation Medicine Department, The Southwest Medical University, Luzhou, Sichuan People’s Republic of China; 3 Department of Physical Therapy, Faculty of Rehabilitation of Kobe International University, Kobe, Japan

**Keywords:** Platelet-rich plasma, Physiotherapy, Rotator cuff injury, Protocol

## Abstract

**Background:**

The study aims to identify whether Platelet-rich plasma (PRP) combined with early physiotherapy has an advantage over PRP alone for rotator cuff injury patients, regarding pain release, function score, tear size, and quality of life improvement.

**Methods:**

This is a single-center prospective non-randomized study implemented in July 2019 at the Affiliated Hospital of Southwest Medical University in Sichuan. Three hundred-forteen patients with rotator cuff injury aged over 18 years were recruited. Participants were assigned to the experiment group (PRP plus physiotherapy) or control group (PRP) by their desire. We used the Constant-Murley score to assess the shoulder function, the Visual Analogue Scale to evaluate shoulder pain, and the MOS Item Short-form Health Survey (SF-12) to measure the quality of life. MRI was applied to measure tear size, and the follow-up duration is 12 months.

**Discussion:**

Our findings will give information on the effects of PRP and physiotherapy on rotator cuff injuries. Physiotherapy might be added to improve the effects of PRP in patients with rotator cuff injuries.

**Trial registration:**

This study was registered in the Chinese clinical trial registry on September 1st, 2019 (ChiCTR1900025563).

**Supplementary Information:**

The online version contains supplementary material available at 10.1186/s12891-021-04171-2.

## Background

Rotator cuff injury is one of the most common causes of pain and shoulder dysfunction, affecting daily living activities, such as brushing hair and getting dressed [1]. The incidence of rotator cuff injury is ranged from 7 to 30% in the general population and increased with age [2]. The mechanism of rotator cuff injury could be divided into two types. Acute injury often caused directly by trauma, and chronic pathology occurred secondary to tendon degeneration, impingement, and repetitive overhead activities [1]. Tear size might progress over time as well as tendon retraction, and muscle atrophy would happen in untreated rotator cuff injury [3]. The above factors might deteriorate the shoulder condition and eventually, decrease the quality of life and health status.

The healing power of nature is fickle due to limited blood supply around the native tendons [4]. Surgery is a common approach to managing injured tendons. The effects were affected by age, history of smoking, and other primary diseases [5], and the re-tear rate is high [6]. Kukkonen et al. [7] suggested that conservative approaches could be the primary treatment for non-traumatic rotator cuff injury. At present, more and more researchers focus on regenerative medicine. Platelet-rich plasma (PRP), rich in a high concentration of platelets, could release many biologically active growth factors through degranulation. These growth factors can induce mitosis, extracellular matrix production, new blood vessel formation, cell maturation, and cell differentiation [8]. The safety and effectiveness of PRP were proved by animal experiments [9, 10]. Besides, a part of clinical studies showed the advantages of PRP [11, 12]. While current systematic review and meta-analyses indicated that PRP seems not superior to other treatments for patients with rotator cuff injury [13, 14]. Clinically, we have not observed the effects of PRP injection therapy alone for rotator cuff injury patients, whether a rehabilitation program is needed to enhance the therapeutic effects of PRP.

To our best knowledge, musculoskeletal physiotherapy, including manual therapies and exercises, seems to be the most supported nonsurgical management for patients with rotator cuff injury [15]. Joint mobilizations, a component of manual therapy, can reduce pain by stimulating peripheral mechanical receptors, suppressing nociceptors, and increasing synovial fluid nutrition [16], and realign collagen, increase fiber slip, reduce adhesion, and restore normal glenohumeral joint kinematics [17]. Moreover, shoulder proprioception, mobility, and stability will be established through exercise training [18]. A recent Cochrane review showed that manual therapy and exercise have identical effects on corticosteroid injections and subacromial decompression approaches [19]. Study suggested that the effects of PRP in addition to eccentric training were superior to eccentric training alone for chronic Achilles tendinopathy [20]. At present, only simple exercises were applied after PRP injection for rotator cuff injury patients [21, 22], and no standardized rehabilitation programs were used after PRP injection.

From the above, the synergistic effect remains unknown. Animal experiments showed that early rehabilitation exercise could enhance the role of PRP in promoting cartilage repair [23]. Jo CH et al. found that PRP injection after arthroscopic repair can improve rotator cuff healing quality. Under this premise, rehabilitation training can reduce the rotator cuff re-tear rate [24]. The result indicated that PRP might have a synergistic effect with physiotherapy. Given that both PRP and physiotherapy have positive effects for rotator cuff injury patients. Our study aims to identify whether PRP combined with early physiotherapy has an advantage over PRP alone for rotator cuff injury patients, regarding pain release, function score, tear size, and quality of life improvement.

## Methods

### Study design

A single-center, prospective non-randomized study with 12 months follow-up duration was designed. The study has started in July 2019 and is ongoing. Three hundred-forteen patients with rotator cuff injury aged over 18 are enrolled and divided into two groups. The participants need to visit a physical therapist 2 days before PRP injection, 1 months, 3 months, 6 months, and 12 months after rehabilitation programs. The feasibility and preciseness of the whole process are supervised by 1 to 2 supervisors.

### Study setting and participants

**Fig. 1:**
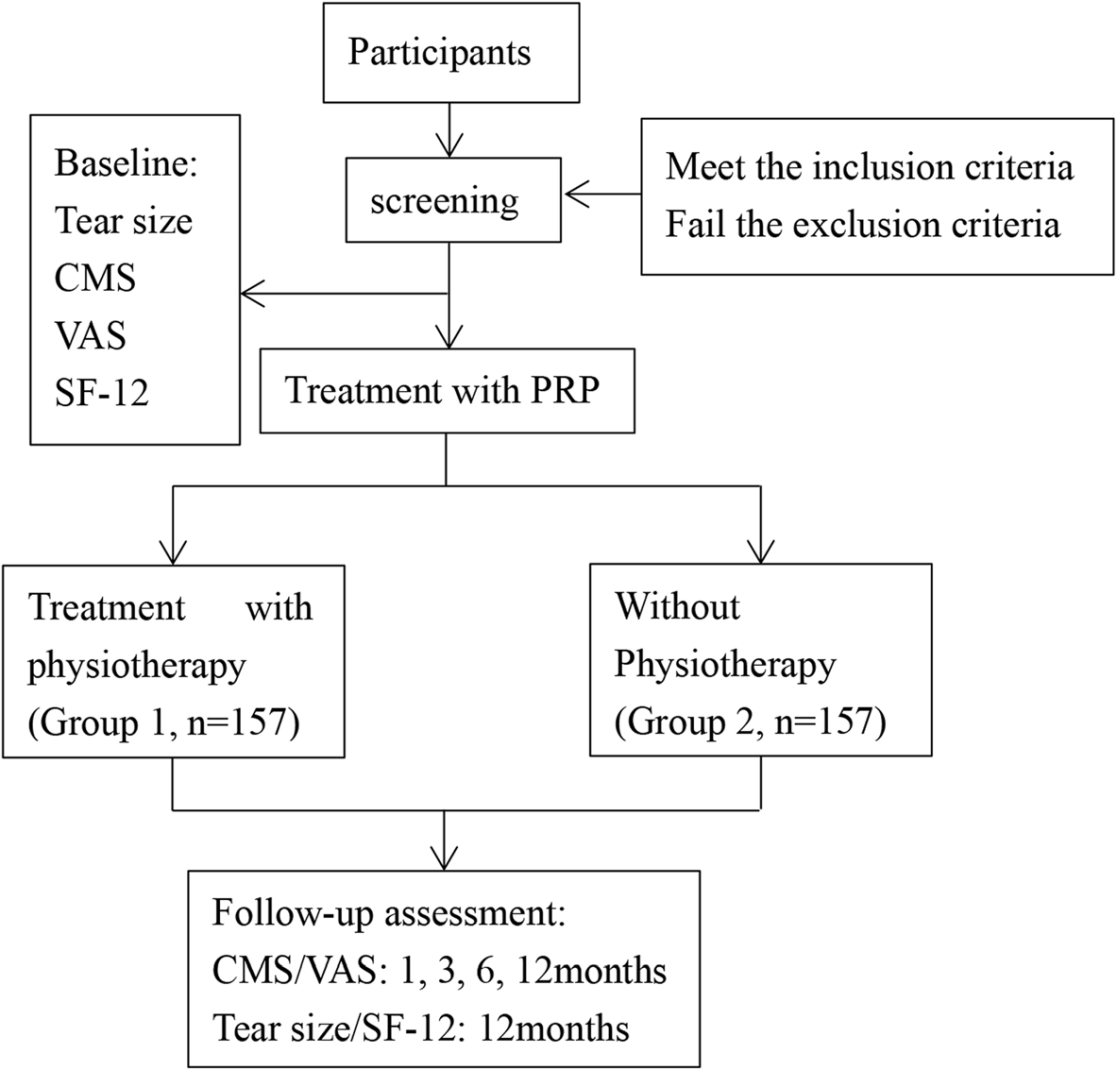
the flow chart of the protocol

This study is being performed in the Affiliated Hospital of Southwest Medical University in China, and all participants are recruited from inpatient and outpatient. All patients were screened rigorously according to inclusion and exclusion criteria. Physicians decide whether the patient is enrolled, and they will tell eligible participants the research protocol in detail. Every eligible participant will be asked to sign a written consent before taking part in this study. The participants voluntarily choose to receive rehabilitation programs after PRP injection, then PRP in addition to physiotherapy group and PRP group were formed. The trial protocol is shown in Fig. 1.

### Ethical issues

This study was registered in the Chinese clinical trial registry (ChiCTR1900025563).

Besides, the current study has received ethical approval from the committee of The Affiliated Hospital of Southwest Medical University (KY2019075).

### Participants

The inclusion criteria are as follows: aged over 18; shoulder pain for at least three months; Partial-thickness supraspinatus or subscapularis muscle injuries were evaluated by clinical examination, and proved by magnetic resonance imaging (MRI). The exclusion criteria are as follows: complete rupture of the rotator cuff; prior shock wave therapy or corticosteroid injection; frozen shoulder; malignancy or bleeding disorders; pregnancy; nerve-related symptoms; inflammatory arthritis; and calcifying tendinopathies.

### MRI

InteraAchieva 3.0 T MRI produced by Philips will be applied in this study. A professional musculoskeletal radiologist identified Partial-thickness rotator cuff tears. The scanning field of view is (FOV) 22 cm × 22 cm, matrix 256 × 256, and the scanning thickness is 3 mm with a 1 mm gap between valid cuts. We will obtain T1- and proton density-weighted fat-saturated images (axial, sagittal, and coronal) and T2- and proton density-weighted fat-saturated images (axial, sagittal, and coronal). Rotator cuff AP tear size will be measured on the sagittal T2-weighted images at anterior and posterior position [25]. The MRI will be performed before PRP injection and 12 months post-treatment.

### PRP preparation and application

All included participants in both groups underwent PRP injection procedure. Peripheral blood (10-15 ml) samples will be collected from all patients based on sterile conditions. Subsequently, storing in an anticoagulation tube with sodium citrate and shaking gently to mix anticoagulation with whole blood. The whole blood will be centrifuged for 10 min at 1000 rpm, and three layers will be observed in this process, upper plasma layer, middle buffy coat layer, and lower red blood cell layer, respectively. We have to draw the plasma and blood platelet into a new sterile tube, and re-centrifuged at 3000 rpm for 10 min, approximately 2-3 ml PRP will be yielded for injection eventually. The injection procedure will abide by the standardized aseptic operation. One mL of 1% lidocaine will be injected by a 25-gauge needle before PRP to anesthetize the rotator cuff. For a while, PRP will be injected into injured tendons under real-time ultrasound guidance. After injection, the patients should lay supine under close watch for 15 min. Only once PRP injection will be performed in the whole treatment.

### Physiotherapy protocol

Physiotherapy will be conducted 2 days after PRP injection in the experiment group. Manual therapy, including joint mobilization, soft tissue massage, and manipulation will be implemented for three weeks, three sessions/week. Meanwhile, supervised exercise therapy in the hospital third a week for three weeks. Home exercise will be carried out once a day and last for three months. The experiment group consists of six exercises: stretching program (crossover arm stretch, sleeper stretch, and anterior shoulder stretch), posture adjustment (scapular retraction, scapular shrug), and strengthening exercise (bilateral external rotation). For crossover arm stretch, patients sit on a chair with good posture, uninvolved side holds the upper arm of the affected arm and pull it across the chest as far as they can; sleeper stretch, patients lie on the affected shoulder side with shoulder and elbow flexion at 90^o^, then use unaffected side to push it down. Hold in the position when patients feel tight in the upper back of the shoulder; anterior shoulder stretch, patients should stand by the door with shoulder abduction and elbow flexion, put the palm on the door, subsequently, lean forward until patients feel a stretch in the front of the affected shoulder; scapular retraction, patients should lie on stomach on a bed with the affected arm hanging over the side, and keep they elbow straight by squeezing shoulder blade toward the opposite side, afterwards, return slowly and repeat; scapular shrug, the participants should abduct shoulder at 10 ^o^ − 20 ^o^ with the palm facing forward, and then shrug shoulder with resistance band; bilateral external rotation, an exercise for rotator cuff and scapula stabilizers, patients stand against the wall holding a resistance band by two hands with shoulder abduction 0^o^ and elbow flexion 90^o^, later, pull the band as far as possible without unnecessary movement. The details of six exercises are listed in Additional file 1: Appendix A.

Pendulum exercise is needed when patients feel uncomfortable after doing the above exercises. Home exercises will depend on the shoulder condition. Each stretching exercise should perform for 30s, and repeat four to five times per day. Scapular retraction is allowed two sets of 10 repetitions with a weight from zero increase to a maximum of three kilograms. The scapular shrug load should start within 0.5 kg to 2 kg, and will be implemented 2 sets of 15 repetitions. The bilateral external rotation will be performed three sets of 10 repetitions and progress to 15 repetitions. Each patient should record their completion daily on a therapeutic form.

### Outcome measures

#### Primary outcomes

The primary outcomes of our study are the Constant-Murley score (CMS) and visual analog scale (VAS). The shoulder function of the patients will be measured by adopting CMS. CMS is a self and examiner based tool, including pain, the activity of daily life, range of motion, and muscle strength [26]. The total score is 100 and divide into four levels, excellent (< 11), good (11–20), fair (21–30), and poor (> 30) [27]. The overall shoulder pain will be assessed through a VAS, with a score of 0 (no pain) to 10 (maximal pain).

#### Secondary outcomes

Tear size change assessed by MRI, the quality of life evaluated by Medical Outcomes Study Short Form 12-Item (SF-12), and adverse events will be recorded as secondary outcomes. An independent radiologist will assess tear size through MRI before PRP injection and 12 months follow-up. The SF-12 is a 12-item questionnaire, focusing on physical functioning, social functioning, bodily pain, vitality, role physical, role emotional, general health, and mental health [28]. In addition, any adverse events caused by PRP injection and physiotherapy will be recorded via self-report. The outcome indicators will be assessed at five different times by a blinded physiotherapist (Table 1).

**Table 1:** Schedules for follow-up assessments and data collection

Assessments	Baseline (Two days after PRP injection)	Finish manual therapy			
		1 month	3 months	6 months	12 months
CMS	√	√	√	√	√
VAS	√	√	√	√	√
SF-12	√				√
Adverse effects	√	√	√	√	√
Tear size	√				√

### Data management

All the involved participants were coded with a number. We used a case report form (CRF) to record the information of participants, including basic information, shoulder function scores, MRI results, and follow-up. The follow-up duration of each participant will be at least 1 year. We also assessed the shoulder function at four other points, two days after PRP injection, 1 month, 3 months, and 6 months. When participants come back to our hospital for a check, the data will be collected by a blinded physiotherapist, other researchers will be forbidden from obtaining the research data. Besides, statisticians will be blinded in our study.

### Sample size calculation

We used the Constant-Murley score serving as a basis of estimating sample size. Based on the study of Sham et al. [12] and our pilot observations, we consider that the change of Constant score could be 8 with a standard deviation of 20. We calculate that 131 participates for each group will be required with alpha set at 0.05 and a power of 0.8. Considering the 20% drop rate, we will recruit 314 participates in total.

### Statistical analysis

All statistical analyses will be performed using SPSS 25.0 (IBM Corporation, Armonk, NY). Continuous variables are described as means and standard deviation. We will use independent *t* tests or Mann-Whitney *U* tests to compare 2 groups. If needed, the stratified analysis will be conducted based on the age of participants and the PRP injection site. A *P* value < .05 will be defined as statistically significant.

## Discussion

Rotator cuff injury is a common issue and can lead to serious shoulder dysfunction, thereby increasing the social burden [29]. PRP could promote tendon healing, and the goal of physiotherapy is to improve shoulder pain and dysfunction. Both PRP injection and physiotherapy could be applied in patients with rotator cuff injury. There is no definitive conclusion as to whether the combination is effective. We first combined PRP with physiotherapy for patients with rotator cuff injury, and we hypothesis that PRP injection in addition to early physiotherapy has an advantage over PRP injection alone. A prospective non-randomized study involving 314 participants will be implemented.

In recent years, PRP has become more and more popular in treating various musculoskeletal diseases due to the remarkable healing augmentation without severe side effects [30]. Several studies reported that PRP as a conservative approach to treat rotator cuff injury. Rha et al. supposed that PRP is safe and effective for rotator cuff disease [31]. Ilhanli I et al. showed that PRP might be as effective as physical therapy [32], PRP in addition to sodium hyaluronate have better clinical outcomes than PRP or sodium hyaluronate alone [11]. However, a high-level trial suggested that PRP cannot enhance tendon healing and improve clinical scores [33]. Most of the studies did not mention rehabilitation program after PRP injection; up to exercises were added [21, 22]. We have to continue exploring the effectiveness of PRP injection due to the evidence is low. Physiotherapy seems as a routine approach for rotator cuff diseases. Whether early physiotherapy could enhance the effects of PRP is unknown. A recent study showed that exercise can increase the concentration of platelets in PRP products [34]. This could be a theoretical foundation for our research. Meanwhile, the rehabilitation protocol, including exercise type, time, and treatment length is various [29]. A standardized rehabilitation protocol after PRP injection is needed.

Some limitations should be listed. First, the current study design is a non-randomized study. The evidence level is relatively low. Considering the feasibility and the actual situation, we select non-randomized study with a large sample size to clarify the correlation between PRP and physiotherapy. In our study, the participants have the right to receive physiotherapy or not. Second, it is impossible to blind therapists. We need therapists to protect participants. Except for limitations, we also have some strengths. This is the first study to analyze the PRP injection combined with physiotherapy for patients with rotator cuff injury. The study design is relatively humanized, and the results are objective. In addition, statistics experts will participate in this study to make the results more reliable. Finally, we can easily establish a clinical database based on this study.

## Supplementary Information

**Additional file 1:.**

## Data Availability

The datasets used and/or analysed during the current study are available from the corresponding author on reasonable request.

## Abbreviations

PRP: Platelet-rich plasma
CMS: Constant-Murley score
VAS: Visual analog scale
SF-12: The MOS Item Short-form Health Survey
