# Supplementary material for: The effect of physiotherapy in rotator cuff injury patients with platelet-rich plasma: study protocol of a non-randomized controlled trial
Source: BMC Musculoskelet Disord. 2021 Mar 20;22:292. doi: 10.1186/s12891-021-04171-2 (PMC7981950; doi:10.1186/s12891-021-04171-2)
Supplement: Supplementary file 1 — Additional file 1:. [file 12891_2021_4171_MOESM1_ESM.pdf]

Stretching program

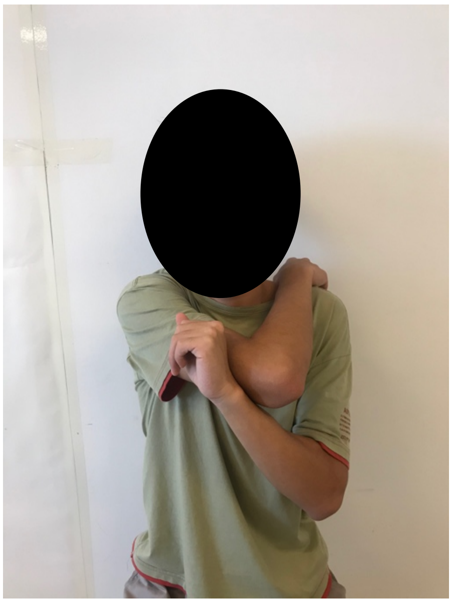

Crossover arm stretch

Hold for 30s, and 4-5 reps in total

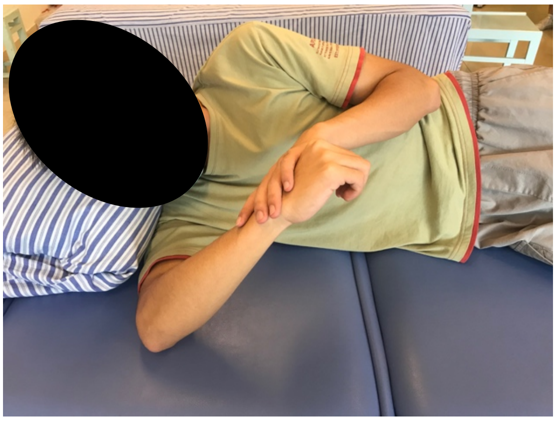

Sleeper stretch

Hold for 30s, and 4-5 reps in total

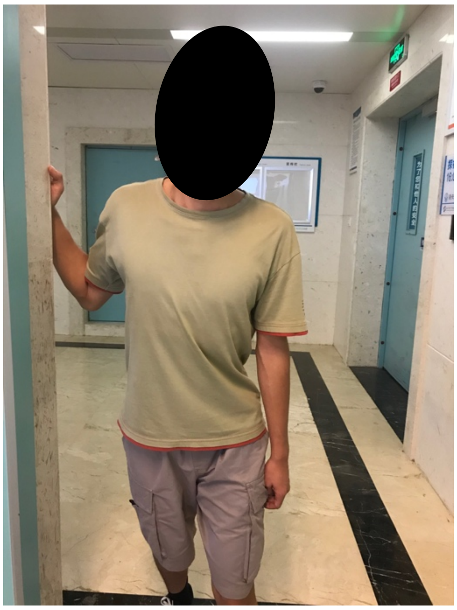

Anterior shoulder stretch

Hold for 30s, and 4-5 reps in total

Posture adjustment

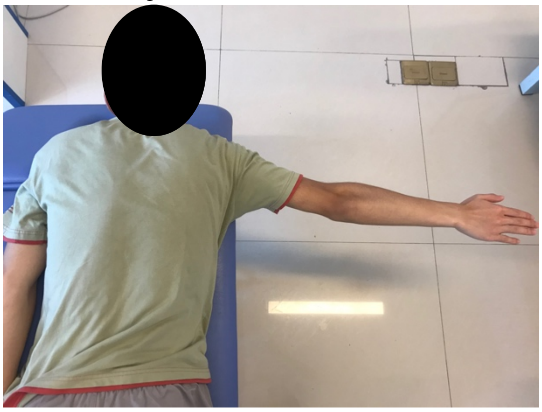

Scapular retraction

Hold for 10-15s, 10 reps and 2 sets with load from 0 to 3kg.

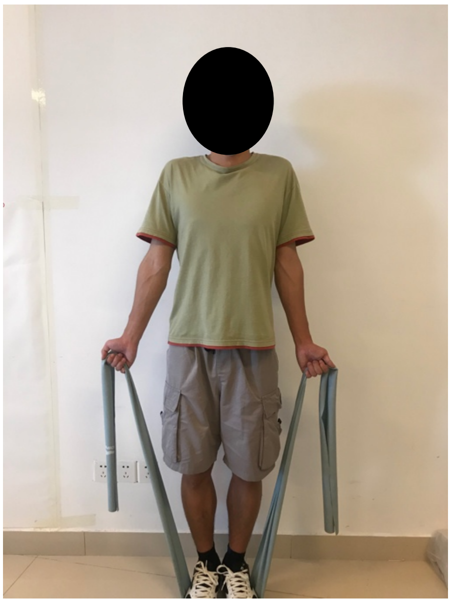

Scapular shrug

Hold for 10-15s, 15 reps and 2 sets with load from 0.5 to 2kg.

Strengthening exercise

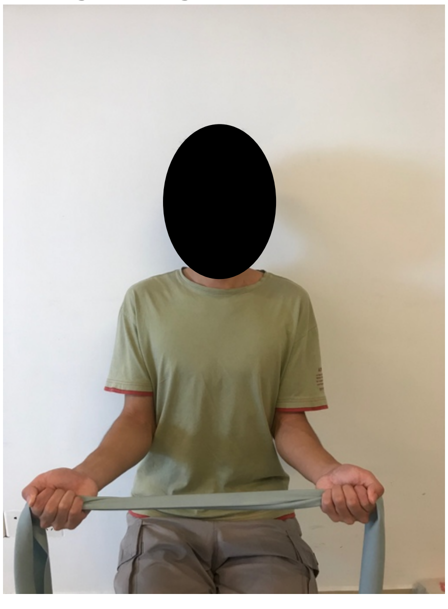

Bilateral external rotation

Hold for 10-15s, 10-15 reps and 3 sets.
